# The Antitumor Activity of Piplartine: A Review

**DOI:** 10.3390/ph16091246

**Published:** 2023-09-04

**Authors:** Allana Brunna S. Duarte, Rebeca C. Gomes, Vitória Regina V. Nunes, Juan Carlos R. Gonçalves, Camylla A. Correia, Ana Zulmira G. dos Santos, Damião P. de Sousa

**Affiliations:** Departament of Pharmaceutical Sciences, Federal University of Paraíba, João Pessoa 58051-970, PB, Brazil; allanabrunna@gmail.com (A.B.S.D.); vitoriareginaventura@gmail.com (R.C.G.); rebecagomes123456@gmail.com (V.R.V.N.); juan.ufpb@gmail.com (J.C.R.G.); camyllacorreia123@gmail.com (C.A.C.); ana.santos@estudantes.ufpb.br (A.Z.G.d.S.)

**Keywords:** natural products, metabolites, medicinal plants, drug, anticancer, cytotoxic, *Piper*, alkaloid, phenylpropanoid, piperlongumine

## Abstract

Cancer is a worldwide health problem with high mortality in children and adults, making searching for novel bioactive compounds with potential use in cancer treatment essential. Piplartine, also known as piperlongumine, is an alkamide isolated from *Piper longum* Linn, with relevant therapeutic potential. Therefore, this review covered research on the antitumor activity of piplartine, and the studies reported herein confirm the antitumor properties of piplartine and highlight its possible application as an anticancer agent against various types of tumors. The evidence found serves as a reference for advancing mechanistic research on this metabolite and preparing synthetic derivatives or analogs with better antitumor activity in order to develop new drug candidates.

## 1. Introduction

Cancer is one of the leading causes of death worldwide and affects roughly 9.6 million individuals annually. The number of cancer cases and deaths is estimated to increase as populations grow, age, and have lifestyles that amplify cancer risk [1,2,3]. Recent global cancer statistics reported an overall of 19.3 million new cases and more than 10 million deaths in 2020 alone, and 28.4 million of new cases expected for 2040, an increase of 47% [4,5]. Available cancer treatments include surgical intervention, radiation, and chemotherapy, which often induce toxicity, resulting in adverse effects that, added to the resistance acquired by malignant cells to chemotherapeutic agents, justifies the search for new effective and low-toxicity compounds [6,7,8].

Natural products are an important source of new drugs and prototypes for synthesizing potential structural analogs. In fact, more than 75% of antitumor drugs approved by the FDA are natural compounds or their synthetic derivatives [9]. Despite having revolutionized cancer chemotherapy, natural-based drugs, such as paclitaxel, vinca alkaloids (vincristine, vinblastine), irinotecan, or etoposide, are not exempt from toxic effects [10]. And it is precisely the lack of further studies on efficacy, safety, solubility, stability, targeting, and toxicity profiles that have hindered the emergence of new drugs [11,12].

In the past decade, several natural biosynthesized metabolites with antitumor properties have been studied and whose pharmacological and clinical application have demonstrated their relevance in the search for new chemical agents for cancer treatment [13]. However, almost none have been approved for clinical use or even advanced from preclinical studies to clinical trials [14]. This illustrates the need for further studies to improve the knowledge of the safety and efficacy of new candidate anticancer drugs.

**Figure 1:**
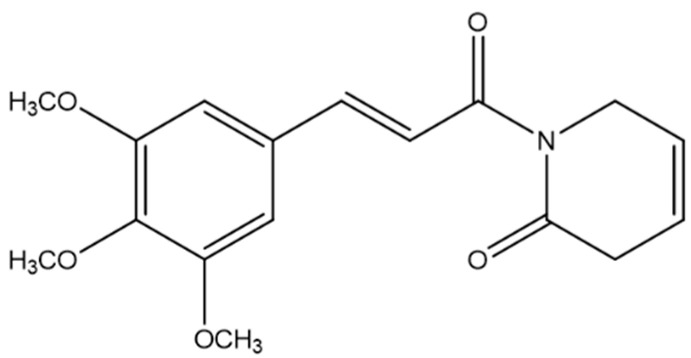
The chemical structure of piplartine.

Piplartine (PL) (Figure 1), also known as piperlongumine, an alkamide obtained from the roots of *Piper longum* L., has been widely studied for its potent antitumor activity against various types of tumor cells, inducing cell death via several pathways, such as apoptosis, necrosis, necroptosis, pyroptosis, oxidative stress, genotoxicity, and autophagy [15,16,17]. Hence, this study conducted an updated review of the antitumor activity of PL and its possible mechanisms of action.

## 2. Results and Discussion

### 2.1. Antiproliferative Activity

Cancer cells have mutations that promote unrestrained cell proliferation. Compounds with the ability to inhibit these processes in antitumor screenings can be selected to investigate their potential as anticancer drug candidates [18,19,20,21]. So, in studies carried out with PL, it was evidenced that this compound inhibits the growth of tumor cells. Meegan et al. [22], in their experiments, investigated the action that PL promoted regarding the proliferation of tumor cells. Thus, the in vitro experiment proceeded with the use of breast cancer cells (MCF-7 cells) and Jurkat T-lymphocytes, where the researchers analyzed the chemical structure of the piperidinone ring and the biological activity against these cell types. For the study, the experimental methodology adopted included the synthesis of PL analog compounds, with some alterations regarding the chemical structure of the initial molecule, modifying certain substituents of the trimethoxyphenyl ring, such as the addition of methoxy groups at specific positions, which optimized the antiproliferative activity of the compound. In this context, the synthesis of PL analog compounds proceeded in two steps, using 5,6-dihydropyridin-2(1H)-one as an intermediate of the reaction. Subsequently, it was tested with the cells, and it was observed that PL and its synthesized derivatives inhibited the proliferation of oncogenic MCF-7 cells at concentrations of 3.4 and 9.6 μM. Additionally, the researchers observed in the experiment a series of cellular events that contributed to apoptosis, such as tubulin depolymerization, destruction of the microtubule cytoskeleton, increased reactive oxygen species (ROS), interruption of the cell cycle in the G2/M phase, and reduction in pro-apoptotic proteins, such as Bcl-2 and Mcl-1.

Furthermore, Niu et al. [23] investigated the effects of PL on the proliferation of cervical-cancer-derived cancer cells. The experiments were divided into five stages. Initially, Hela cells were treated with PL at different concentrations, and cell viability was assessed using MTT assay. Next, specific antibodies were used in Hela cells to localize target proteins. Subsequently, a cellular transport assay was conducted to monitor CRM1-mediated nucleocytoplasmic transport using GFP proteins (Foxo1-GFP and NES-GFP). Additionally, during the experiment, immunoprecipitation, Western blotting, and mass spectrometric analysis were performed. In this context, the researchers observed that the half-maximal inhibitory concentration (IC_50_) was 16.3 μM after 24 h of PL exposure. The researchers also noted that PL promotes the export of CRM1 in cancer cells, leading to the accumulation of Foxo1, p21, p53, and IκB-α in the nucleus. These proteins are tumor suppressors and consequently contribute to the inhibition of cancer cell proliferation. This scenario suggests that PL possesses a mechanism of action that involves modulating the passage of compounds that transit to the cell nucleus.

Wang et al. [24] investigated the effect of PL on the retinoblastoma cancer cells, HXO-RB44 cells. They created drug-resistant cell lines, rendering the cells resistant to chemotherapy. The research group then tested the oncogenic cell line with PL alone and in combination with chemotherapy drugs. Subsequently, isolated administrations of 10 or 20 μM of PL showed moderate and dose-dependent effects on cancer cell growth. The authors also evaluated the sensitivity of each cell clone to its respective drug when co-administered with 10 or 20 μM of PL, and the IC_50_ values of each drug against the resistant cells were lower than in the control group (no PL). The results showed that PL exhibits a moderate dose-dependent activity and restores sensitivity to chemotherapy. Furthermore, PL promoted a series of cellular events, including intracellular drug accumulation, cell cycle arrest, and induction of apoptosis. Additionally, proteins contributing to chemotherapy resistance had their cytoplasmic levels reduced. PL also regulated the PI3K/AKT and PKCζ signaling pathways, which are involved in cell nuclear communication with the extracellular environment. These pathways are associated with cell growth, proliferation, and programmed cell death. Overall, the experiments suggest that PL has the potential to reverse chemotherapy resistance in retinoblastoma cancer cells in this in vitro assay.

Lewis et al. [25] conducted a study using the non-small-cell lung cancer (NSCLC) cells, A549 cells. The study aimed to investigate the inhibitory effects of the molecules C188-9 and PL on the STAT3 signaling pathway of NSCLC cells, which is relevant for the survival of NSCLC cells. Initially, the experiment involved eight NSCLC cell lines and one non-cancerous human bronchial cell line. Next, for the C188-9 inhibitor, variable IC50 values were obtained in the range of 3.06 to 52.44 μM, while for PL, the values were 0.86 to 11.66 μM. Thus, the experiment observed that the inhibitors reduced pSTAT3 levels in all cell lines and induced apoptosis by reducing mRNA levels of STAT3 genes related to apoptosis resistance. Additionally, in mouse tumor xenograft models, it was observed that PL and C188-9 reduced tumor growth and decreased mRNA levels of genes related to the STAT3 pathway. Therefore, the results obtained in the study suggest a fundamental connection between the STAT3 pathway and the survival of NSCLC cells. Furthermore, they also indicate that the PL and C188-9 inhibitors have potential in treating this cancer.

In the study conducted by Song et al. [26], 3-(4,5-dimethylthiazol-2-yl)-2,5-diphenyltetrazolium bromide (MTT) cell proliferation assay demonstrated that PL suppresses cell proliferation of gastric cancer (GC) cells and MKN45 cells in a concentration- and time-dependent manner. Flow cytometry was used to analyze the cell cycle distribution of MKN45 and AGS cells treated with different concentrations of PL for 24 h. PL reduces STAT3 activity by negatively regulating JAK1/2 activity in gastric cancer cells and has also been shown to modulate the expression of STAT3-dependent tumor-associated genes [26].

Karki et al. [27] treated several cell lines derived from pancreatic cancer (Panc1, L3.6pL), kidney cancer (786-O), lung cancer (A549), and breast cancer (SKBR3) with 5 or 10 μM of PL for 24 and 48 h. Subsequently, they observed that PL treatment triggers a decrease in the expression of the transcription factors Sp1, Sp3, and Sp4 in the cells. This implies that a reduction in these factors promotes cellular alterations, as they regulate genes that are correlated with oncogenic events. Additionally, in the study, PL induced programmed cell death, and it was observed that the apoptotic effects of PL are attenuated after treating the cells with glutathione (GSH), which is a cellular antioxidant. This suggests that the pro-apoptotic action of PL occurs through an increase in reactive oxygen species (ROS), and when the cells were treated with the antioxidant, it resulted in a reduction in PL activity. Subsequently, xenotransplantation of tumors in mice was performed, and it was evident that tumor growth and weight regressed as PL reduced the transcription factors Sp and consequently the genes regulated by these factors.

In a study conducted by Harshbarger et al. [28], structural and biochemical analyses revealed the inhibition mechanism of glutathione S-transferase Pi 1 (GSTP1) by PL, using cells from various types of cancer, including HeLa (cervical cancer), SW620 (colorectal adenocarcinoma), and PANC-1 (pancreatic carcinoma) cell lines. After treatment with PL, the IC_50_ values were determined, with HeLa cancer cells showing a value of 5.8 μM, SW620 cells showing 7.9 μM, and PANC-1 cells showing 17 μM. Initially, the study used X-ray crystallography to analyze the molecular interactions of PL with the enzyme GSTP1. The data suggest a model in which PL is a prodrug whose intracellular hydrolysis initiates the formation of the hPL–GSH conjugate, which blocks the active site and inhibits GSTP1, consequently inhibiting the proliferation of cancer cells [28].

In their experiments, Niu et al. [29] investigated the effects of PL on activated B-cell receptor (ABC) diffuse large B-cell lymphoma (DLBCL) cells, including the OCI-Ly10, U2932, and DB cell lines. The study aimed to explore the potential of PL in selectively suppressing these tumor cells, and it was observed that 2 μM inhibited 40% of the cells. The results demonstrated that PL selectively inhibits ABC-DLBCL cells by blocking the nuclear translocation and phosphorylation of the p65 subunit, leading to the inhibition of NF-κB activation. Additionally, PL induced dose-dependent apoptosis in this specific cell line. Intracellular changes were also observed in cancer cells, including the suppression of anti-apoptotic proteins and an increase in pro-apoptotic protein expression. These findings suggest a promising new strategy that could serve as an alternative treatment approach for ABC-DLBCL with activated B-cell receptors [29].

In the experiments conducted by Thongsook et al. [30], cell growth was suppressed after PL treatment in all cell lines (human cholangiocarcinoma (CCA) cell lines KKU-055, KKU-100, KKU-139, KKU-213, and KKU-214 and two immortalized cell lines MMNK-1 and NIH3T3) in a dose-dependent manner (0, 2.5, 5, 10, and 15 µM) for 48 h. PL inhibited growth and induced apoptosis in most cell lines, except KKU-100 and NIH3T3, via activation of the caspase pathway, which are proteins involved in the process of programmed cell death. Furthermore, Western blot analysis showed a dose-dependent increase in p-JNK and p-ERK in KKU-055 (5, 10, and 20 µM) and KKU-100 (10 and 20 µM) cells at 1 h after PL treatment. Next, the association between reactive oxygen species (ROS) elevation and JNK-ERK activation in PL-induced CCA apoptosis was confirmed. Additionally, PL increased the expression level of p-Akt and the downstream effector of Akt, Bad, only in KKU-100 cells in a dose-dependent manner and positively regulated the anti-apoptotic protein Bcl-2. In summary, the experiment indicates that PL shows antitumor activity in the tested cells, promoting the inhibition of cell proliferation and the activation of cellular pathways and events that trigger apoptosis.

In this context, Liu et al. [31] demonstrated that PL significantly diminishes cell survival in three bladder cancer cell lines (T24, BIU-87, and EJ) in a concentration-dependent manner (2.5, 5, 10, and 20 μM for 24 h), with IC_50_ in the range of 10–20 μM. Flow cytometry analysis showed an increase in the percentage of cells in the G2/M phase from 10% to 27% and a reduction in the percentage of cells in the G1/G0 phase from 48% to 30% after treatment with 20 μM of PL, indicating G2/M phase arrest. Importantly, PL suppressed T24 cell movement, indicating a significant effect in inhibiting cancer metastasis. Additionally, PL at concentrations of 5, 10, and 20 μM significantly reduced T24 cell migration and invasion within 24 h of treatment. The treatment also resulted in a dose-dependent decrease in the number of cells with protrusions. Furthermore, the fluorescence intensities of DCFH-DA in cells (T24 and BIU-87) treated with PL at 5, 10, and 20 μM also increased, indicating ROS accumulation. The study also revealed that PL mainly hinders bladder cancer cell migration and invasion through ROS, Erk, and PKC signaling pathways. Treatment with PL (1.5 or 3.5 mg/kg-day) in an animal model mimicking bladder cancer for 14 days showed a significant reduction in tumor weight and volume. Moreover, when analyzing the expression of molecules involved in the epithelial–mesenchymal transition (EMT), PL at a concentration of 3.5 mg/kg-day clearly demonstrated a significant reduction in the expression of EMT-associated molecules—Slug, β-catenin, ZEB1, N-Cadherin, Claudin-1, and ZO-1—in the cancer xenografts [31]. 

In another study, cancer cell growth was inhibited in the presence of PL. Han et al. [32] conducted an in vitro experiment and found that at micromolar concentrations of PL (IC_50_ = 2.8 × 8.5 μM), there was significant inhibition of growth and survival in two Epstein–Barr-virus-positive Burkitt lymphoma cell lines (Daudi and Raji) and two Epstein–Barr-virus-negative Burkitt lymphoma cell lines (Ramos and DG-75). However, this concentration did not affect peripheral blood B lymphocytes, which remained unaffected. Moreover, PL-dependent cytotoxicity was partially affected by reducing NF-κB and MYC activity, with the former caused by inhibiting IκBα degradation, nuclear translocation of p65, and binding of NF-κB dimers to DNA sequences in gene promoters. The study indicates that PL induces carcinoma cell death by targeting the cells’ “non-oncogene co-dependency” on elevated antioxidant defense pathways, which develop from an imbalance between ROS formation and the cells’ neutralization capacity induced by the transformation of normal cells into cancer cells. The findings in the study suggest that treatment with PL inhibits NF-B/MYC, triggering the death of Burkitt lymphoma cells. Furthermore, PL induces changes in the expression of E2F1, MYB, and GADD45B genes, which are NF-B/MYC target genes involved in the survival of cancer cells. Therefore, the study proposes that PL treatment disrupts the genes involved in maintaining the survival of oncogenic cells and subsequently triggers programmed cell death in Burkitt lymphoma cells [32].

Similar findings related to the tumor cell inhibitory activity of PL were also evidenced by Zheng et al. [33], who found that PL treatment (2.5–5 mg/kg) in xenograft mice suppressed the dose-dependent tumor growth of non-small-cell lung cancer (NSCLC). Furthermore, the expression level of active caspase-3 increased, while p50 expression decreased in xenograft tissues of nude mice. Additionally, the DNA-binding activity of NF-κB decreased in a dose-dependent manner (2.5–5 mg/kg) in lung tumor tissues, and the nuclear expression of p50 and p65 also decreased. Additional findings showed that Fas and DR4 expression and cleaved caspase-3, cleaved caspase-8, and Bax expression increases, while Bcl-2 expression decreases in a dose-dependent manner [25]. In summary, in both in vitro and in vivo experiments, PL reduced tumor cell growth by targeting the suppression of transcription factors involved in cell survival, such as NF-κB. Additionally, the increased expression of caspase-3 progressed toward a mechanism of programmed cell death. Thus, these findings indicate that PL may be a potential option for the treatment of lung cancer.

In another study, Bezerra et al. [34] demonstrated IC_50_ values of 0.8 and 0.7 μg/mL for PL in in vitro tests on SF-295 (glioblastoma) and HCT-8 (colon carcinoma) cell lines, respectively. The authors used 5-FU as an active control and found IC_50_ values of 0.3 and 0.2 μg/mL for SF-295 and HCT-8 cell lines, respectively. Subsequently, in vivo tests were conducted using an HFA model, which differed from traditional xenograft models by having a shorter duration and requiring a smaller quantity of compounds. In this stage, PL and the crude extract were tested. For this purpose, cells were cultured in biocompatible and semipermeable hollow fiber models and then subcutaneously implanted into the dorsal region of BALB/c nude mice. After treatment with PL, tumor cell proliferation of SF-295 (62.2% and 61.5%) and HCT-8 (33.7% and 50.8%) cells reduced at doses of 50 and 100 mg/kg/day. As for the crude extract of *P. tuberculatum*, tumor reduction ranged from 24.6% to 54.8%. Finally, 5-FU reduced tumor cell proliferation of SF-295 (53.7%) and HCT-8 (70.0%) cells at doses of 20 mg/kg/day. Overall, both PL and the crude extract showed efficacy in both experiments, suggesting an activity that promotes tumor suppression.

Similarly, Zou et al. [35] investigated the effects of PL on human gastric cancer cells and normal cells. The tests revealed that PL administration in cancer cells inhibits cell proliferation in a dose-dependent manner. However, in healthy cells, it showed a minimal effect. This led to the discovery that PL treatment triggers a series of cellular events, such as the accumulation of ROS in cancer cells, contributing to apoptosis progression. The experiment also demonstrated that PL has the ability to interact with the enzyme TrxR1, leading to its inactivation. Consequently, this increased ROS levels in the cell, leading to endoplasmic reticulum stress and ultimately apoptosis. Moreover, caspase-9 activation and the modulation of Bcl-2 proteins were observed. Subsequently, in vivo studies were conducted using xenotransplant models of SGC-7901 cells in immunodeficient mice. Intraperitoneal doses of PL at 4 and 12 mg/kg for 15 days significantly reduced the volume and weight of the SGC-7901 tumor compared to the vehicle control. In summary, the study demonstrated that PL selectively inhibits cancer cells in both in vivo and in vitro settings through cellular mechanisms involving increased ROS, enzyme inactivation, and modulation of proteins involved in cancer signaling pathways [35].

In contrast, Ryu et al. [36] observed that PL shows cytotoxicity in HepG2 cell growth at 5 mM of PL, while over 80% of HepG2 cells survived at 2 mM. In this scenario, the researchers noticed that PL increases the phosphorylation of AMPK and acetyl-CoA carboxylase (ACC), and these data were obtained by using the compound C (6-[4-(2-piperidin-1-yl-ethoxy)-phenyl)]-3-pyridin-4-yl-pyrrazolo [1,5-a]-pyrimidine), an AMPK inhibitor, in part of the experiment. When cells were treated with the AMPK inhibitor, phosphorylation did not occur. However, in cells not treated with the inhibitor, AMPK phosphorylation increased in the presence of PL. Thus, phosphorylated AMPK promotes fatty acid metabolism, while concurrently inhibiting its biosynthesis [36].

Hu et al. [37] revealed that PL tested in osteosarcoma (OS) cells, from U2OS and MG63 cell lines, presented an IC_50_ of 10.02 and 8.38 μM, respectively. Additionally, cell migration and invasion in OS cells (U2OS and MG-63) were negatively regulated after treatment with PL (at concentrations of 2.5, 5, and 10 μM). PL also dose-dependently attenuated the levels of Vimentin and N-cadherin, while increasing E-cadherin expression in both U2OS and MG-63 cells. Furthermore, as the PL dose increased, the mRNA expression of SOCS3 also increased, despite the suppression of JAK2 and STAT3 phosphorylation. Additionally, the researchers found that miR-30d-5p is a regulator contributing to OS development. In this regard, treatment with the same concentrations of PL negatively regulated miR-30d-5p, which was associated with reduced luciferase activity of SOCS3-WT, inhibiting the JAK2/STAT3 signaling pathway mechanism, thereby directing the reduction in tumor cell proliferation. These promising results suggest the potential of PL against OS. Finally, in an in vivo xenotransplant tumor model in immunodeficient mice, treatment with 2.5, 5, or 10 μmol/kg of PL suppressed the tumor volume and weight. Moreover, miR-30d-5p levels and JAK2/p-STAT3 activation were negatively regulated, while SOCS3 expression significantly increased in tumor tissues [37].

Lu et al. [38] investigated the effect of PL on non-small-cell lung cancer (NSCLC) cells using the NSCLC cell lines A549, H1299, H520, and SPC-A-1. The researchers reported that A549 cells, after treatment with PL, showed 74 differentially expressed miRNAs with alterations of more than 3 times, of which 27 were upregulated and 47 were downregulated. MiR-34b-3p showed a 14-fold increase in changes in the group treated with 10 μM of PL. Furthermore, CCK-8 assay showed that both positive regulation of miR-34b-3p and PL treatment inhibited cell proliferation, with 10 μM of PL promoting a significant apoptotic effect. When comparing the effects of positively regulated miR-34b-3p and PL on tumor proliferation in vivo, a xenotransplantation experiment was performed in nude mice, revealing that the tumor volume and weight were significantly reduced in mice inoculated with the miR-34b-3p group and the group treated with 5 mg/kg of PL. Additionally, Western blot analysis showed that treatment with PL alone significantly decreases the expression of the TGFBR1 protein. Moreover, the study observed that PL promotes an increase in miR-34b-3p, which negatively regulates the TGFBR1 protein, thus generating antitumoral effects. This is because the overexpression of the TGFBR1 protein contributes to migration and invasion [38].

However, Rawat et al. [39] revealed that the cytotoxicity of PL inhibits the proliferation of intestinal cancer cells, INT-407 and HCT-116, in a concentration- and time-dependent manner. The IC_50_ of PL for INT-407 cells was 13 and 9 μM, while for HCT-116 cells, it was 8 and 6 μM after 24 and 48 h of incubation, respectively. Furthermore, 8 μM (IC_50_ for HCT-116), 13 μM (IC_50_ for INT-407), and 20 μM of PL promoted increased fragmentation and deformation of the cell nuclei, indicating that PL can induce morphological changes, chromatin condensation, and nuclear fragmentation. Additionally, PL at the same concentrations listed before elevated intracellular ROS levels, which may lead to lethal oxidative stress, mitochondrial dysfunction, and nuclear fragmentation. Notably, P53, P21, Bax, and SMAD4 were significantly upregulated after PL treatment, while BCL2 and SURVIVIN were downregulated. In summary, PL demonstrated potential cytotoxicity in the study and represents a promising alternative.

Chen et al. [40] demonstrated that treatment of oral cancer cells, known as oral cavity squamous cell carcinoma, of the SAS and CGHNC8 lineages with 2.5 and 5.0 µM of PL reduced the number of tumor spheres by approximately 67.28% and 91.58%, respectively, with 5 µM of PL inducing the formation of small spheres with diameters of 48.27 µM and 95.62 µM in SAS and CGHNC8 cells, respectively. After treatment with 5.0 µM of PL, the mRNA levels of SOX2, NANOG, and Oct-4 decreased to 42%, 60%, and 36% in SAS cells, and 86%, 96%, and 87% in CGHNC8 cells, respectively. Additionally, PL treatment increased CK18 expression levels by 1.6- and 1.8-fold in SAS and CGHNC8 cells, respectively. Similarly, PL reduced Oct-4, NANOG, and SOX2 protein levels; increased CK18 protein levels; and reduced the cell migration and invasion ability of both cell lines in a concentration-dependent manner. Furthermore, compared to control cells, PL-treated cells exhibited higher E-cadherin expression levels and decreased N-cadherin and vimentin expression levels. Moreover, the radiation sensitivity levels of SAS and CGHNC8 cells treated with 0.625 µM of PL and radiation increased by 47.5% and 25.63%, respectively. Treatment with PL (5 µM) for 48 h reduced the growth of SAS and CGHNC8 cells to 11.2% and 21.9%, respectively; PL also significantly suppressed colony formation, and only a handful of colonies were formed after treatment with a concentration of 2.5 µM. Investigating the effects of PL on tumor growth in vivo, SAS xenograft tumors were established in BALB/c nude mice, and analyses showed that treatment with 2.4 mg/kg of PL promoted the slowest tumor growth, and by day 42, tumor growth decreased by 63%. Finally, this treatment also revealed that the tumor weight was reduced by 66% in the PL-treated group. The study also revealed that PL inhibits the ability of cancer stem cells to form and suppresses the expression of the transcription factors SOX2, POU class 5 homeobox 1, and Nanog homeobox. However, it increased the expression of the differentiation marker cytokeratin. PL also suppressed cell migration and invasion, eliminating the epithelial–mesenchymal transition; PL also increased chemo- and radiosensitivity and suppressed tumor growth in vitro and in vivo [40].

In this context, the experiments of Allaman-Pillet et al. [41] demonstrated that 10 μM of PL decreases the growth of retinoblastoma cells from WERI-Rb and Y79 cell lines by 2-fold and 3-fold, respectively. It is worth noting that WERI-Rb cells treated with PL exhibited some classic signs of apoptosis, such as caspase-3 activation and subsequent PARP cleavage. However, regarding PL-induced Y79 cell death, it appears to be caspase independent, as no caspase-3 activation and subsequent PARP cleavage could be measured. Treatment of WERI-Rb and Y79 cells with 10 μM of PL demonstrated higher reactive oxygen species (ROS) production. Additionally, the mRNA expression of CCNA2, CCNB1, CDC25C, and CDK1 significantly decreased in PL-treated cells. Moreover, PL increased the mRNA content of CDKN1A in WERI-Rb and Y79 cells, while no change in the CDKN1B transcript was observed. Lastly, microarray analysis detected positive FOXM1 regulation in human retinoblastoma cells, and in Y79 retinoblastoma cells, FOXM1 depletion was shown to affect cell invasive ability by targeting MMP2. Overall, PL exhibits cytotoxic effects by elevating ROS levels, promoting cell death through oxidative stress. Additionally, PL modulates the expression of factors involved in cell cycle participation. Therefore, the experiment suggests that the use of PL directs cell death in retinoblastoma cells [41].

Delaney et al. [42] revealed that exposure to 10 µM of PL decreases metabolic activity by at least 50% for all breast-cancer-derived cancer cells from the tested cell lines: MDA-MB-231, BT-549, and Hs578T. Remarkably, MDA-MB-231 cells were relatively resistant to PL and showed little to no growth inhibition in the presence of 1 or 2.5 µM. The study also demonstrated that MDA-MB-231 cells treated with 1 µM of PL exhibit reduced cell motility/invasion. Reduction in MMP2 and MMP9 expression, increased expression of miR-200c, decreased IL-6 synthesis, decreased ZEB1 and Slug expression, and increased E-cadherin expression and changes in cell morphology were also observed; PL also inhibited the induction of ZEB1 by TGFb. Reactive oxygen species accumulated in cells treated with 2.5 µM of PL, while changes in the expression of metastasis-associated genes were abolished by the addition of exogenous glutathione at 10 µM. In the study, administration of the vehicle (DMSO) or PL (2.5 mg/kg) via daily intraperitoneal injection for 9 days in BALB/c mice bearing 4T1 tumors showed that, at the end of treatment, there was no significant difference in the weight of the primary tumor between the vehicle- and PL-treated animals, indicating that the concentration of PL used was not cytotoxic to 4T1 cells in vivo. However, there was a significant reduction in metastasis in PL-treated mice, regardless of the weight of the primary tumor. In summary, the study suggests the potential of PL to selectively induce cell death in cancer cells, while not having a significant effect on normal cells. PL plays roles such as inhibiting EMT, reducing metastatic activity, decreasing IL-6, and increasing intracellular ROS levels, selectively causing cell viability impairment [42].

The research conducted by Zou et al. [43] presented positive data regarding the activity of PL against the proliferative activity of various tested cells. The results addressed the antitumor activity of the molecule in cancer cells, including human glioma U87MG cells, colorectal cancer HCT-116 cells, lung cancer A549 cells, and leukemia lK562 cells, using MTT assay. Initially, the findings in the study showed that PL exhibits inhibitory effects on the tested cancer cells, with IC_50_ values ranging from 5.09 μM to 16.15 μM, depending on the cell type. Thus, the lowest IC_50_ values were observed for colorectal cancer and colon cancer cells, suggesting greater effectiveness against these cell lines. The tests also reported antimigration and anti-invasion activity. Additionally, PL at 50 nM reduced the percentages of HCT-116 cells adhered to HUVECs in a dose-dependent manner. The study determined that treatment with 4 mg/kg of PL in a model of lung metastasis of HCT-116 colon cancer cells in BALB/c mice prevented weight loss in tumor-bearing mice and prolonged their survival [43].

In the study by Kumar and Agnihotri [44], PL exhibited chemopreventive effects in animal models of colon carcinogenesis induced with DMH + DSS. A dose of 3.6 mg/kg of PL significantly reduced the tumor burden and colon tumor size, with normal histopathological evaluation, restoration of the calyceal cell population, nullified adenoma formation, and inflammatory cell infiltration. Moreover, PL demonstrated potent antineoplastic activity against colon cancer cell growth by inhibiting Ras proteins and the PI3K/Akt signaling cascade. The PL-mediated inhibition of tumor cell growth was associated with a reduction in Ras protein levels and their preferred PI3K protein levels, leading to the suppression of Akt/NF-κB, c-Myc, and cyclin D1 activity, which are crucial in cell cycle regulation. Additionally, it was found to interrupt cell cycle progression in the G2/M phase and induce the mitochondrial apoptotic pathway by negatively regulating Bcl-2 levels. Furthermore, PL significantly increased the expression of caspase-3 fragments. Therefore, these findings are highly significant in understanding the mechanisms through which PL exerts its antitumor activity in the colon.

In contrast, Bosc et al. [45] demonstrated that PL inhibits iCP cell lines, with an IC_50_ of 15 μM and an EC_50_ of 2.7 ± 0.1 μM in HeLa cells. The study revealed that PL selectively inhibits the human immunoproteasome in various cancer types, indicating that the immunoproteasome is involved in the survival of cancer cells. It also showed that PL does not significantly inhibit the human constitutive proteasome. Thus, the results suggested that the mechanism for immunoproteasome inhibition, independent of ROS elevation, may also play a role in the anticancer effects observed with PL treatment [45].

Kumar et al. [46] used the experimental mouse model of DMH/DSS-induced colon cancer, demonstrating that 3.6 mg/kg of PL in DMH + DSS-treated animals significantly reduces LDH, LASA, and TSA activity. Furthermore, PL showed protective effects against the detrimental impact of DMH/DSS on calyceal cells, as PL administration in DMH + DSS-treated animals sharply decreased mucin layer erosion as well as the mucin content of the colonic epithelium, in addition to increasing the number of calyceal cells per crypt. Furthermore, PL promoted an approximately 7-fold reduction in phosphorylated NF-κB levels compared to the DMH + DSS group, in addition to higher IκB levels and reduced phosphorylated IKK α/β levels. Moreover, PL suppressed NF-κB COX-2 signaling events and its downstream targets; PL reduced the levels of β-catenin, IL-6, JAK2, and STAT3, the latter discretely, and the compound also decreased Jagged-1 and Notch-1 levels, which are responsible for abrogating Notch signaling. Additionally, PL exhibited strong anti-angiogenic and anti-invasion activity, as it significantly Inhibited the formation of new blood vessels and reduced MMP-9 expression, a component capable of degrading extracellular matrix components. Finally, PL significantly increased, by about 3-fold, E-cadherin levels and decreased by 2-fold N-cadherin levels, also reducing vimentin and TCF/ZEB expression, making it clear that PL can inhibit the EMT pathway [46].

Liu et al. [47] provided evidence that PL treatment (5, 10, 15, and 20 μM) potently induces FOXO3A–GFP nuclear translocation in a dose- and time-dependent manner. Treatment with 15 μM of PL resulted in a significant increase in BimEL isoform protein levels, and real-time PCR analysis revealed significantly higher BIM mRNA levels in HeLa and MCF-7 cells treated with 15 μM of PL. The study also revealed IC_50_ values of 12.89 and 10.77 μM in HeLa (cervical cancer cell line), 13.39 and 11.08 μM in MCF-7 (breast cancer cell line), and 12.55 and 9.725 μM in MGC-803 (gastric cancer cell line) cells at 24 and 48 h, respectively. In contrast, PL treatment had little effect on the cell viability of L02 cells and HUVECs. Western blot analysis showed increased levels of cleaved caspase-9 and cleaved caspase-3 with 15 μM of PL. Depletion of FOXO3A markedly attenuated the 15 μM of PL-induced activation of caspase-9, caspase-3, and BIM. Additionally, 8 mg/kg of PL was administered intraperitoneally in a subcutaneous MCF-7 cell xenograft model in mice, showing a reduction in MCF-7 tumor volume and weight; PL also increased nuclear FOXO3A abundance and decreased phosphorylated FOXO3A protein expression in tumor tissues. Finally, PL significantly suppressed Akt phosphorylation without affecting its overall protein level. These data suggest that FOXO3A dephosphorylation and nuclear accumulation are mediated by Akt inactivation in response to PL activity [47].

**Figure 2:**
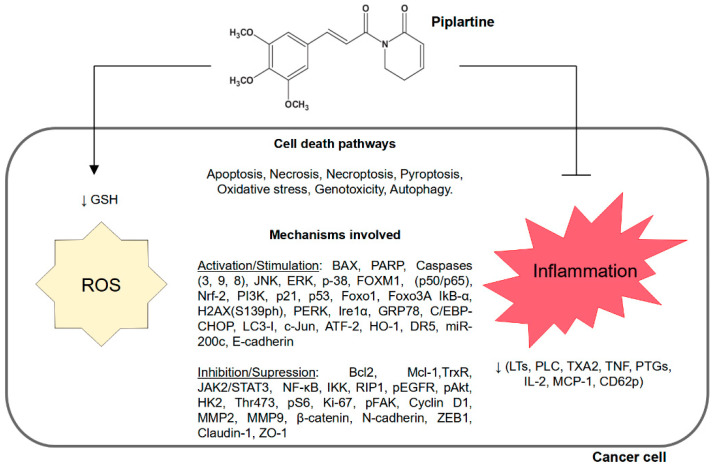
Mechanisms of piplartine in cancer cells.

Song et al. [48] conducted a study to investigate the cytotoxic effect of PL on melanoma cell lines A375, A875, and B16-F10. These cells were treated with different concentrations of PL (0.625, 1.25, 2.5, 5, 10, and 20 mM), and a cytotoxic effect was observed against melanoma cell lines, with concentration dependence at 0.6, 2.5, and 2.5 mM. Notably, colony formation in A375 cells decreased by 52.6, 92.6, and 99.6%, respectively, with 0.6, 1.2, and 2.5 mM of PL, suggesting A375 melanoma growth inhibition. Moreover, after treatment of A375 cells with 2.5 mM of PL for 48 h, cells in the G0/G1 phase reduced from 43.97 to 31.95%, cells in the S phase decreased from 30.49 to 22.88%, and cells in the G2/M phase increased significantly from 25.22 to 44.85%. In A875 cells, nevertheless, an increase in the G2/M phase was also observed. A375 cells treated with PL at 0.6, 1.2, and 2.5 mM showed 84.8, 70.5, and 58.2% of surviving cells, respectively, demonstrating the apoptotic role of PL. Indeed, ROS levels increased by 1.3-, 3.4-, and 5.6-fold after treatment with 0.6, 1.2, and 2.5 mM of PL, respectively. Finally, the relative expression of p21 and p27 increased by 6.3- and 5.7-fold after treatment with 2.5 mM of PL. Similarly, the study proved that PL activates caspase cleavage cascades, including cleaved caspase-3, Bax, and Bcl-2, and increases p-JNK relative expressions [48]; see Figure 2.

### 2.2. Correlation of Increased Intracellular Reactive Oxygen Species and Decreased Inflammation in Cancer Cells with Piplartine

Increased intracellular reactive oxygen species (ROS) can lead to cellular damage (e.g., DNA strand destruction and cell membrane lysis), affect cellular metabolism reactions, and activate certain cell death pathways, such as apoptosis [49,50,51]. Cancer cells are relatively vulnerable to induced oxidative stress compared to normal cells [52]. This hypothesis was also supported by researchers who showed that small molecules, including PL, induce cell death in cancer cells by targeting redox defense systems [53].

In this context, Kim et al. [54] investigated whether PL would increase ROS levels in high-grade glioblastoma (HGG) cells. The researchers observed that PL increases ROS levels after interacting with various redox regulators, such as peroxiredoxin 4 (PRDX4), and selectively kills HGG cells, with little effect on normal neural stem cells (NSCs). This was suggested because HGG cells expressed higher levels of the proposed PL targets than did NSCs. Furthermore, PL exacerbated intracellular ER stress, an effect that was mimicked by suppressing peroxiredoxin 4 expression, which is responsible for detoxifying ROS from the endoplasmic reticulum [54].

Similarly, treating U266 myeloma cells with PL (0.5–1.5 µM) for 30 min resulted in intracellular changes. The effect of PL was reversed with N-acetylcysteine treatment. Primarily, PL triggered a series of events in the cells, such as MYC overexpression, which potentiated the elevation in ROS levels, contributing dramatically to apoptosis and consequently reducing proliferation. Additionally, it was observed that PL indeed induces DNA double-strand breaks, which were further increased by MYC overexpression, suggesting a genotoxic effect of PL. Furthermore, as expected, PL induced higher levels of ROS and superoxide in multiple myeloma (MM) cell lines, with most MM cell lines being sensitive to PL; OPM-2 cells were less sensitive, while U266 cells were the most sensitive to PL [55]. PL significantly induced poly(ADP-ribose) polymerase (PARP) cleavage and increased γ-H2A.X and p21 levels in H929 and OPM-2 MM cells. The effect of arsenic trioxide (ATO), a known ROS inducer, was also tested, and the data revealed that ATO increases ROS/superoxide and γ-H2A.X levels. PL was more potent in growth inhibition than ATO, despite inducing a decrease in ROS and superoxide dismutase levels, suggesting that PL proposes anticancer activities resulting beyond the oxidative stress pathway. Moreover, PL predominantly reduced glutathione (GSH) levels without affecting glutathione reductase activity. However, PL directly interacts with various proteins, including glutathione S-transferase pi 1 (GSTP1), glutathione S-transferase omega 1 (GSTO1), and glyoxalase I (GLO1), which are essential for catalyzing the conjugation of reduced glutathione to electrophilic substances. It was also found that PL treatment results in the cleavage of poly(ADP-ribose) polymerase (PARP), an enzyme involved in cellular repair mechanisms, indicating the activation of DNA damage repair and maintenance mechanisms [55].

In a study, Pei et al. [56] found that PL has a significant inhibitory effect on the leukemic glutathione system, while causing only a limited and transient disruption in normal cells. In this regard, in addition to its effect on glutathione metabolism, PL provided potent cytotoxic effects on acute myeloid leukemia (AML) cells. This preferential effect is closely linked to its selective toxicity against leukemia cells and other types of cancer cells [56]. In another study, researchers found that PL treatment significantly increases ROS production, protein glutathionylation, and Nrf-2 expression in both cell lines [57]. In nude mice with HT29 tumors, PL (7.5 mg/kg daily) decreased the tumor volume by 40% and intratumoral mutant p53 protein levels. The antitumor efficacy of 1,3-bis(2-chloroethyl)-l-nitrosourea (BCNU) or doxorubicin in HT29 colon tumor cells was significantly increased by PL, followed by apoptotic protein expression. These clinically relevant findings suggest that the PL-induced oxidative milieu facilitates poor functional restoration of mutant p53 via protein glutathionylation and contributes to higher drug sensitivity [57].

Han et al. [58] also investigated the effects of PL and found that it inhibits the proliferation of all B-ALL cell lines, although not normal B-cells, in a dose- and time-dependent manner, and induces apoptosis via ROS elevation. In fact, PL did not sensitize most B-ALL cell lines to dexamethasone and increased p21 expression in B-ALL cells via a p53-independent mechanism. Bissinger et al. [59] assessed the effects of erythrocyte exposure to PL (30 µM) for 48 h. In conducting the experiment, they observed that after treatment with PL, there is a significant reduction in forward scatter, suggesting a change in cell morphology, and an increase in annexin V binding, indicating induction of programmed cell death. Another finding was that PL does not significantly modify the intracellular concentration of Ca2+ ([Ca2+]i), and its effect was not dependent on the presence of extracellular Ca2+, indicating that PL exerts its effects independently of pathways controlled by variations in intracellular calcium ion concentration. The data also demonstrated that PL significantly increases the formation of ROS (reactive oxygen species) and the abundance of ceramide [59].

Adams and colleagues [60] demonstrated that PL has an EC_50_ (50% effective concentration) of 2.8 and 7.1 μM in H1703 (lung cancer cell line) and HeLa (cervix cancer cell line) cells, respectively. Furthermore, a 20 μM concentration of PL was demonstrated to induce cancer cell death and increase ROS. In addition to higher ROS levels, 20 μM of PL was found to affect other oxidative stress markers, promoting a 60% depletion in total cellular GSH in a luminescence-based assay for cellular glutathione (GSH/GSSG-Glo) in the EJ bladder carcinoma cell line. Nonetheless, Qian et al. [61] testified that PL shows IC_50_ values of 5.6, 8.4, 6.8, and 8.2 μM in the Bel7402, Bel7402/5-Fu, HepG2, and HGC27 cell lines, respectively. Moreover, the antitumor activity of PL increased ROS production by inhibiting thioredoxin reductase (TrxR) activity; the molecule showed significant inhibitory effects on TrxR in Bel-7402/5-FU cells. Furthermore, 3.0 μM of PL was proven to increase ROS levels by approximately 26.7%; still, PL at doses of 2 or 5 mg/kg significantly suppressed tumor growth in Bel7402/5-Fu cells in mice [61]. The effects of PL and related mechanisms are described in Table 1.

In another study, Thongsom et al. [30] investigated the effects of PL on CCA cell lines and obtained IC_50_ values of 4.2, 5.2, 6.2, 8.8, and 15.9 µM for the CCA cell lines (KKU-055, KKU-213, KKU-214, KKU-139, and KKU-100) and 5.7 and 12.7 µM for two immortalized cell lines (MMNK1 and NIH3T3). In addition, caspase-3 and BCL2-associated X protein (Bax) activation was observed in KKU-055 cells treated with 10 µM of PL, although this effect was not observed in KKU-100 cells. Furthermore, the authors found significant ROS accumulation in KKU-100 cells treated with 20 μM of PL, corroborating the theory that PL induces cell death mediated by ROS. Reactive oxygen species have been reported to participate in apoptosis regulation through extracellular-signal-regulated kinases (ERK), c-Jun N terminal kinases (JNK), and p38 activation; Western blot analysis revealed a dose-dependent increase in p-JNK and p-ERK in KKU-055 (5, 10, and 20 µM) and KKU-100 (10 and 20 µM) cells within 1 h after PL treatment. Furthermore, PL only increased p-Akt, Bad, and Bcl-2 expression in KKU-100 cells in a dose-dependent manner, in addition to increasing G2/M cell populations in KKU-055 (from 36 to 63%) and KKU-214 (from 8 to 49%) cells in a dose-dependent manner (5 and 10 µM). There was, however, no change in the cell cycle of KKU-100 cells after PL treatment. Lastly, 10 µM of PL was demonstrated to suppress and stabilize FOXM1 expression, inhibiting 20S proteasome activity and inducing cell death in cancer cells through PARP and caspase-3 activation [30].

Nan et al. [84] demonstrated in their study that PL induces the depletion of survivin protein levels through the proteasome-dependent pathway mediated by reactive oxygen species (ROS) in vitro, while exerting a remarkable inhibitory influence on the proliferation of cancer cells of the ovary. The results revealed that 10 and 20 μM of PL effectively depletes survivin expression in A2780 and OVCAR-3 cells, corroborating the theory that PL induces survivin reduction in ovarian cancer cells in vitro, which may contribute to its apoptotic effect. It is worth noting that treatment at these concentrations does not alter survivin mRNA expression. Thus, the study found that PL induces survivin depletion at the post-transcriptional level via increased ubiquitin-proteasome degradation. Indeed, in the presence of PL, the IC_50_ value of OVCAR-3 cells was 4.59 μM, which is lower than survivin overexpression, with an IC_50_ of 9.65 μM, suggesting that survivin overexpression increases the ovarian cancer cell survival rate. Furthermore, 20 μM of PL in A2780 cells and 10 μM in OVCAR-3 cells increased ROS, inducing apoptosis, and doses of 20 mg/kg in vivo in a xenograft model of A2780 cells in BALB/c mice decreased the tumor weight and volume. Additionally, survivin significantly decreased in tumors after exposure to PL [84].

Another study supports such a hypothesis; Afolabi et al. [88] evaluated the antitumor and immunomodulatory functions of PL in human skin cell carcinoma A375, human cervical carcinoma Hela, human hepatocellular carcinoma HepG2, and human hepatocarcinoma Huh7 tumor cell lines. This study demonstrated that 2 µM of PL promotes the onset of apoptosis in A375 cells, although only doses of 4 µM or above were enough to induce apoptosis in HeLa cells. HeLa and Huh7 cell lines treated with (1–8 μM of PL) showed induced expression of pro-apoptotic proteins (e.g., pro-caspase-3 and cleaved caspase-3) and inhibited anti-apoptotic protein Bcl-2 in a dose-dependent manner. Moreover, PL inhibited tumor cell growth in a dose-dependent manner (1–8 μM of PL) in A375, HeLa, HepG2, and Huh7 cells. The authors’ findings also revealed that concentrations below 8 µM do not induce apoptosis in normal human cell lines (HPDE, LO-2, HK-2, and MCF10a). Likewise, tumor cells pretreated with 2 µM of PL increased NK cell cytolysis. Indeed, PL was proven to induce intracellular ROS accumulation in a dose-dependent manner (1–8 µM), with 1 µM of PL being enough to significantly increase intracellular ROS in A375 cells compared to 4 and 8 µM of PL required to generate significant intracellular ROS generation in HeLa, HepG2, and Huh7 cells. The researchers’ data also demonstrated that treatment with 2–8 µM of PL significantly induces misfolded protein accumulation in A375, HeLa, and HepG2 cells and 4–8 µM of PL is required for Huh7 cells, which activates autophagy. Nonetheless, PL treatment increases NK and tumor cell conjugation. Lastly, treatment with 10 mg/kg of PL in vivo in RMA-S (MHC-I-deficient tumor cells), B16F10, and CT26 (MHC-I-sufficient) tumors in mice demonstrated growth suppression and tumor weight alteration. With this in mind, it was plausible that PL suppressed tumor growth in an NK-cell-dependent manner, as it increased NK cell activation in vivo [88]. Qian et al. [62] synthesized 27 PL analogs and evaluated their antiproliferative activity. The results demonstrated that PL has an IC_50_ of 3.72, 4.4, 3.2, 1.67, and 7.85 μM in estrogen-receptor-positive (MCF-7) and triple-negative breast cancer (MDA-MB231) cells, human lung cancer (A549) cells, HeLa (cervical cancer) cells, KB (keratin-forming HeLa) cells, and KB-VIN (vincristine-resistant KB) cells.

Moreover, PL possessed inhibitory activity against TrxR in MDA-MB-231 cells with an IC_50_ level of 1.52 μM, thereby demonstrating that its antiproliferative property primarily results from TrxR inhibition—just as PL acts by decreasing TrxR1 protein expression and increases in a dose-dependent manner (0.01–10 μM) ROS levels. Additionally, PL induced apoptosis in MDA-MB-231 cells at a concentration of 1.8 μM, such as lower anti-apoptotic Bcl-2 and Bcl-xL expression; this concentration also induced autophagosome and autolysosome accumulation in MDA-MB-231 cells (e.g., increased LC3-II and Beclin-1 expression). The analyses also showed that 10 mg/kg of PL in BALB/c mice inoculated subcutaneously with MDA-MB-231 cells reduced tumor volume and mass [62].

According to Meng et al. [63], a series of PL derivatives were synthesized and their in vitro and in vivo pharmacological properties were evaluated. PL has an IC_50_ of 6.53, 5.31, 8.15, 6.25, and 7.02 μM in MDA-MB-231, A549, HGC27, HT-29, and HepG2 cell lines, respectively. In addition, PL showed an IC_50_ of 1.52 μM, corresponding to the inhibition of TrxR activity in MDA-MB-231 cells. A dose-dependent increase (0.01–5 μM) in ROS levels was also observed in MDA-MB-231 cells. Furthermore, a concentration of 400 nM slightly reduced cell migration. The study also found that treatment with 10 mg/kg PL in BALB/c mouse models inoculated with MDA-MB-231 cells decreased the tumor volume and weight by 45% [63].

Yamaguchi et al. [64] reported that PL alone induces the ferroptotic death of pancreatic cancer cells and that combined treatment with PL plus CN-A and/or a lower dose of sulfasalazine remarkably enhances PL-induced ferroptosis. This study revealed that PL increases ROS levels in a dose-dependent manner in pancreatic cancer PANC-1 and MIAPaCa-2 cells. In cells treated with 7.5 μM of PL, the population of ROS-positive cells did not increase, whereas 15 μM of PL increased this population by roughly 13%. It is worth noting that 14 μM of PL decreased cell viability by 10% in the control group. Furthermore, 8 μM of PL reduced the cell viability of the MIAPaCa-2 cell line by approximately 70%, just as PL induced cancer cell death by inducing ferroptosis. Furthermore, PL decreased mouse embryonic fibroblast (MEF) viability in a mild dose-dependent manner, although such viability was >60% in the presence of PL, even at a higher concentration (8 μM) [64].

Wang et al. [65] synthesized a series of 17 new PL derivatives and evaluated their pharmacological properties. The results demonstrated that PL has an IC_50_ of 7.53, 5.79, 4.68, and 6.61 μM in human gastric carcinoma cell lines HGC27 and SGC7901, human breast carcinoma MCF7 cells, and colon carcinoma HCT116 cells, respectively. In addition, PL showed an IC_50_ of 1.76 μM due to TrxR activity inhibition in HGC27 cells, and it also increased ROS levels as concentrations increased (0.01–10 μM), with a 10% induction occurring at a concentration of 10 μM. The authors also observed a reduction in mitochondrial transmembrane potential in HGC27 cells as concentrations increased (0.01–10 μM). Analysis data also showed that PL increases the amount of G2/M phase cells at concentrations of 0.8 and 3 μM. Nevertheless, 4 μM of PL showed apoptotic activity in HGC27 cells, which was confirmed by upregulating Bax expression and PARP cleavage and negatively regulating Bcl-2 expression in HGC27 cells. Lastly, the study also provided evidence of increased H2AX(S139ph), p53, and p-p53 expression [65].

In another study, Wang et al. [66] examined the radiosensitizing effect of PL in colorectal cancer cells under both aerobic and hypoxic conditions, and to unveil the mechanisms, the activity of GSH and Trx systems, ROS production, ROS-induced DNA damage, cell cycle arrest, and the oxygen consumption rate of tumor cells were assessed. This research demonstrated that PL treatment in colorectal cancer cells (CT26 and DLD-1) led to an IC_50_ of 15.98 and 11.20 μM, respectively. Their findings revealed that apoptosis and necrosis increased to 6.2 and 9.9% and 12.9 and 18.7% at 15 μM in CT26 and DLD-L cells, respectively. First, 15 and 10 µM of PL caused excessive ROS production due to glutathione depletion, reaching 45 and 40% inhibition for CT26 and DLD-1, respectively; this also occurred due to TrxR inhibition by 35 and 30% for CT26 and DLD-1, respectively. Second, 15 and 10 μM of PL increased the intrinsic and hypoxic radiosensitivity of tumor cells, which was linked to ROS-mediated increases in DNA damage, G2/M cell cycle arrest, and cell respiration inhibition—in the latter case from 5 and 10 μM of PL for CT26 and DLD-1 cells. Finally, the radiosensitizing effect of PL at 2.4 kg/mg/day was verified in vivo in CT26-tumor-bearing mice. Additionally, PL enhanced the tumor response to single and fractionated radiation, which is associated with antioxidant system inhibition, significantly increasing the tumor-bearing mice’s survival rate. However, it was ineffective on its own [66].

Yan et al. [67] investigated the antitumor action of PL using in vitro experiments and observed an IC_50_ of PL in A549 (human lung adenocarcinoma) cells of 10.17 ± 0.18 µM; in HepG2 (human hepatocellular carcinoma) cells, it corresponded to 8.08 ± 0.30 µM; in HT-1080 (human fibrosarcoma) cells, it was 5.55 ± 0.66 µM; and in WI-38 (human lung fibroblasts) cells, it was >60 µM. Thus, the authors found that PL increases ROS levels and exhibits clear time- and dose-dependent TrxR inhibitory activity, thereby proving to be a potent TrxR inhibitor with an IC_50_ value below 10 μM for a 2 h incubation period. Hence, TrxR is one of the targets by which ROS generation is promoted (O_2_—and H_2_O_2_), resulting in selective A549 cell death. Moreover, data showed that a concentration of 20 μM of PL only produces a marginal effect on inducing apoptosis and cell cycle arrest [67].

Duan et al. [89] examined the anticancer effects of PL on gastric cancer cells both in vitro and in vivo and further investigated the underlying mechanisms. Our data demonstrated in vivo that PL treatment using concentrations of up to 10 μM for 2 weeks remarkably reduces the number and size of cell colonies in a dose-dependent manner compared to untreated cells. Increasing PL doses induced G2/M phase arrest, which was accompanied by higher GADD45α and p21 protein levels, while a decrease in cyclin B1 and cdc2 protein levels was also observed; in addition, the authors also found increased apoptosis, which was partially related to caspase-3 and caspase-7 inhibition and caspase-9 activation, PARP inhibition, and significantly increased intracellular ROS levels. The authors’ findings led them to believe that PL increases intracellular ROS levels in gastric cancers in a STAT3-independent manner, although the detailed mechanism is still elusive and must be further investigated. Additionally, profound antitumor effects were observed in the PL-treated group (3.6 mg/kg bw per day) without apparent toxicity compared to the control group [89].

Huang et al. [68] illustrated how the use of specific tools to quantitatively measure and manipulate intracellular GSH and H_2_O_2_ can contribute to a refined understanding of the involvement of these species in the action of chemotherapeutic agents, such as PL. In addition, they performed in vitro analyses and observed that in the case of PL, the concentration required to inhibit the tumor cell population by 50% is 4.4 ± 0.1 μM in HeLa and 11.7 ± 2.0 μM in A549 cells. HeLa cells exhibited a 4-fold decrease in GSH levels in response to PL application; in comparison, A549 cells showed virtually no GSH depletion effects. Comparing the level of Prx-2 dimerization (the presence of this Prx-2 dimer evidences the existence of H_2_O_2_, which is derived from oxidative stress) in HeLa cells versus A549 cells showed that HeLa cells are much more susceptible to elevated H_2_O_2_ than A549 cells in response to PL application. The study suggested that the cells’ ability to prevent H_2_O_2_ accumulation may be important for PL resistance [68]

In this context, Chen et al. [69] examined the anticancer effects of PL on HCC cells in vitro and in vivo and further investigated the underlying mechanisms. The in vitro experiments performed demonstrated that PL has a cytotoxic effect selectively in HCC cells but not in normal hepatocytes, with an IC_50_ of 10–20 μM, while significantly lower concentrations only suppress HCC cell migration/invasion. Furthermore, PL selectively increased ROS in HCC cells, which subsequently activated or positively regulated PERK/Ire1α/GRP78, p38/JNK/Erk and C/EBP homologous protein (CHOP) [69]. It was also found that treatment with PL or annexin-A1-mimicking peptide reduces cell proliferation and viability and modulates the expression of the chemokine MCP-1, cytokine IL-8, and genes involved in inflammatory processes. The results also point to an inhibitory effect of PL on tubulin expression [91].

PL blocked, in a dose-dependent manner, collagen-induced platelet aggregation, calcium influx, CD62p expression, and thrombus formation in collagen with a maximum inhibition at 100 μM, and it also reduced collagen-induced platelet microvesiculation. Moreover, PL blocked JAK2 and STAT3 activation in collagen-stimulated platelets, and this inhibitory effect significantly decreased in platelets pretreated with a STAT3 inhibitor. Despite PL inducing ROS production in platelets, quenching ROS using excessive reducing agents, 20 μM GSH and 0.5 mM L-cysteine, did not block the inhibitory effects. The NADPH oxidase inhibitor apocynin also had no effect [92].

Han et al. [70] sought to analyze other mechanisms through which PL exerts its antitumor effects at 10 μmol/L IC_50_. The researchers found that PL blocks NF-κB activated by TNFα and several other cancer promoters. This negative regulation was accompanied by phosphorylation inhibition and IκBα degradation. Further investigation revealed that this alkaloid directly interacts with IκBα kinase (IKK) and inhibits its activity; IKK inhibition occurred through an interaction with its cysteine 179, as mutation of this residue to alanine abolished PL activity. Indeed, NF-κB inhibition negatively regulated the expression of proteins involved in cell survival (Bcl-2, Bcl-xL, c-IAP-1, c-IAP-2, and survivin), proliferation (c-Myc and cyclin D1), inflammation (COX-2 and IL-6), and invasion (ICAM-1, -9, CXCR-4, and VEGF) [70].

### 2.3. Activation of Cell Death Pathways Using Piplartine

Apoptosis induction by PL in cells has been observed in numerous experiments, highlighting a certain selectivity in cancer cells exposed to low concentrations. However, even at higher concentrations, a more significant resistance in the process of activation of cell death pathways in healthy cells has been obtained. In this context, Roh et al. [71] evaluated the effect of PL alone and in combination with cisplatin in human head and neck cancer (HNC) cells and normal cells by measuring growth, death, cell cycle progression, reactive oxygen species (ROS) production, and protein expression and in tumor xenograft mouse models. PL in vitro induced death in cancer cells, while the viability of normal cells was only minimally affected at the highest concentration (15 μM). Furthermore, Western blot analyses showed that 10 μM of PL significantly increases the expression of wild-type p53, of the pro-apoptotic p53 targets PARP and PUMA, and of p21 in AMC-HN9 cells. Pro-apoptotic protein levels also increased in AMC-HN3 cells expressing mutant p53 (R282W) and in UMSCC-1 p53 null cancer cells. The authors described that PL exposure at 10 μM for 1 and 3 h significantly increases ROS levels in head and neck cancer (HNC) cells, in addition to decreasing GSH levels and increasing GSSG levels [71].

Fofaria et al. [72] established the role of STAT3 in vitro and in vivo in anoikis resistance in pancreatic cancer. This research also conducted an in vitro experiment with PL (5–10 µM) and reported that anoikis resistance sharply decreases in all pancreatic cancer cell lines (AsPc-1, Panc-1, HPAC, L3.6PL, and COLO-357). Most cell lines decreased by roughly 90% in anoikis resistance after treatment with 10 µM of PL. At the same time, a significant and concentration-dependent decrease was observed in STAT3 phosphorylation, in addition to Mcl-1 and Bcl-2 expression, which are anti-apoptotic proteins transcriptionally regulated by STAT3. Thus, the researchers hypothesized that STAT3 is critical in conferring anoikis resistance to pancreatic cancer cells. In fact, the authors found that PL negatively regulates STAT3 protein levels in all cell lines tested. They observed marked downregulation of Mcl-1 and Bcl-2, the anti-apoptotic proteins, which are under transcriptional regulation by STAT3. Furthermore, after PL treatment, massive PARP cleavage occurred, indicating anoikis induction. Subsequently, in vivo studies using mice injected with untreated Panc-1-like cells showed significantly higher metastasis levels in the lungs and liver compared to the PL-treated group; in addition, the PL-treated cells showed a smaller average tumor volume than their untreated counterparts [72].

Bullova et al. [17] found that the levels of cleaved apoptosis markers caspase-3 and cleaved PARP after PL treatment in MPC cells significantly increase in vivo. The measured increase in apoptosis markers depended on the dose and time (1–10 μM for 24 h) used in the experiment. The results also showed that concentrations of 5 and 10 μM of PL decrease cleaved RIP1 levels, whereas ROS levels increased at higher PL concentrations after 3 h of treatment. In parallel, MPC cells exhibited lower viability in hypoxia when exposed to 5 μM of PL after 24 h or a concentration between 5 and 10 μM after 48 h. The authors also observed necrosome assembly in cells treated with PL for 24h at 5 μM of PL in normoxia. These results were suggested as a result of the activation of ERK-1/2, p38α, and JNK1/2/3 pathways, all known transducers of ROS-associated signaling [17].

Moreover, the in vivo tests showed that mice treated with PL (24 mg/kg/day) or the vehicle for 28 days showed significantly reduced tumor growth from the first week of treatment. In addition, tumor growth remained low until the end of the study. Liver metastases were found at a similar frequency in both groups, and in the treated group, the number of lung metastases was significantly lower than in the control group (44% vs. 90%). Additional metastases were also found in the peritoneum or close to the primary tumors in 80% of the untreated mice, although this number only reached 22% in the treated mice. Elevated necrosome levels were also observed in treated animals, suggesting a necroptosis-inducing effect of PL in vivo and higher ROS levels in the treated group, suggesting that PL exerts its cytotoxic effects via ROS induction in vivo [17].

In this context, Liu et al. [90] investigated whether PL can also be effective in killing GBM cells selectively through ROS-dependent mechanisms and observed that a PL concentration of 20 μM induces cell death in vivo in three glioblastoma multiforme cell lines (LN229, U87, and 8MG) but not astrocytes in the cultures. Furthermore, PL concentrations reasonably increased ROS and reduced glutathione levels in LN229 and U87 cells. Notably, the signaling pathways that participated in PL-induced cancer cell death remained elusive, although an early and prominent activation of JNK and p38, two typical ROS response pathways, was observed in glioblastoma multiforme cells exposed to PL [90].

Similar findings correlating apoptotic activity in tumor cells as a result of PL administration were reported by Zheng et al. [33], who observed that PL induces apoptotic cell death in vitro and suppresses the DNA-binding activity of NF-κB in a concentration-dependent manner (0–15 μM) in non-small-cell lung cancer (NSCLC) cells [33]. In contrast, Faria et al. [73] observed that treatment with PL for 48 h reduces cell proliferation in vitro in medulloblastoma cells at 5 μM. When normal human brain cells (hf5281) were incubated with PL, there was little reduction in cell proliferation, even at the highest PL concentration (10 μM). Furthermore, animals with detectable signals were treated with a subcutaneous injection with PL (50 mg/kg, daily for 2 weeks), and a marked reduction in medulloblastoma growth was observed in PL-treated mice compared to the DMSO-treated controls [73].

Wang et al. [74] conducted in vitro experiments and described the relationship between PL and autophagy. The researchers found that 20 µM of PL significantly suppresses U937 cell proliferation (leukemic cell type) within 24 h, while 10 or 20 µM of PL considerably suppressed U937 cell proliferation within 48 h. Furthermore, 10 or 20 µM of PL was shown to activate autophagy (as measured using LC3-targeted fluorescence microscopy) in U937 cells in contrast to U937 cells incubated with 0 µM. In addition, LC3-I protein expression was induced by 0–20 µM of PL in a concentration-dependent manner. In particular, 10 or 20 µM of PL significantly increased LC3-I protein expression in U937 cells compared to U937 cells incubated with 0 µM. In contrast, 10 or 20 µM significantly decreased the p-Akt/Akt ratio in U937 cells compared to U937 cells incubated with 0 µM of PL. The results also demonstrated that treatment with 10 or 20 µM significantly inhibited p-mTOR protein expression in U937 cells compared to the 0 µM group. In fact, treatment with 10 or 20 µM of PL significantly induced p-p38 protein expression in U937 cells compared to U937 cells incubated with 0 µM. Lastly, the researchers found that treatment with 10 or 20 µM of PL significantly increases caspase-3 activity in U937 cells compared to U937 cells incubated with 0 µM [74].

Zhang et al. [75] tested PL against five human osteosarcoma cell lines (A549, HCT-116, MDA-MB-231, SK-Hep-1, and Saos-2) and observed that PL shows IC_50_ values 6.84, 7.34, 10.6, 13.3, 9.49, and 7.31 µM, respectively. In addition, PL induced apoptosis of HCT-116 and Saos-2 cell lines. For Saos-2 cells, the percentage of apoptotic cells after treatment with 1 and 5 µM was 26 and 33%, respectively. For HCT-116 cells, the percentage of apoptotic cells after treatment with 1 and 5 µM was approximately 10% [75]. In this context, Okamoto et al. [76] observed that only the HR-deficient cell lineage including *brca1^−/−^* (between 0.8 and 1.2 μM of PL) and *brca2^tr/−^* (between 1.2 and 1.6 μM of PL) shows hypersensitivity to PL in vitro. These data suggest that PL induces DNA double-strand breaks [76].

A similar study by Lee et al. [77] explored the PL-induced selective killing of cancer cells, with a special focus on HO-1, and reported the possible mechanism involved in PL-mediated Nrf2 activation and HO-1 induction. They observed that in addition to PL promoting cell death, the molecule is selective with cancer cells. The maximum selective killing effects of PL on cancer cells were observed at 5 μM, particularly in MCF-7 cells, although there was only a marginal effect on MCF-10A cell viability. In this context, Wu et al. [78] also observed the selectivity of cell death, although the concentrations obtained were for PL that showed an IC_50_ of 22.85, 6.04, 5.86, 8.46, and 35.04 μM in A549 (human lung carcinoma), HCT116 (human colorectal carcinoma), ZR-75-30 (human breast carcinoma), MDA-MB-231 (human breast carcinoma), and MRC-5 cells, respectively [78].

A study by Randhawa et al. [79] tried to identify the intracellular signaling mechanisms by which PL leads to increased cell death in colon cancer. The results indicated that PL induces a concentration- and time-dependent decrease in the viability of HT-29 and HCT 116 cells, with an IC_50_ value of 10.1 μM in HT-29 cells and 6.4 μM in HCT-116 cells at 48 h, albeit it only inhibited the growth of normal NCM460 colon mucosal cells at 10 μM after 48 h. Additionally, total cell counts determined by trypan blue exclusion assay in HT-29 cells treated with different concentrations of PL (5–40 μM) for 24, 48, and 72 h demonstrated that PL inhibits proliferation and induces cell death in HT-29 cells; the results also showed that PL induces apoptosis in HT-29 colon cancer cells. Maximum increases in p-ERK expression were observed at the lowest concentrations (2.5–10 μM) of PL, whereas higher concentrations (20–40 μM) only produced slightly higher p-ERK levels [79].

Yao et al. [80] found out whether PL has antitumor activity in multiple myeloma (MM) cells and reported that incubation with PL for 48 h inhibits MM cell growth in a dose-dependent manner, with IC_50_ values ranging from 1 to 5 μM. Treatment with different concentrations of PL for 24, 48, or 72 h also inhibited NCI-H929 cell growth in a dose- and time-dependent manner. PL increased apoptosis in time- and dose-dependent ways, as measured by the cleavage and activity of caspase-3, caspase-9, and caspase-8. In addition, PL decreased anti-apoptotic protein Bcl-2 levels and increased the Bax/Bcl-2 ratio in NCI-H929 cells. These data suggest that PL induces MM cell apoptosis via Fas- and mitochondria-dependent pathways. PL can also inhibit the growth and survival of MM cells by altering the microenvironment of the bone marrow (BM) [80].

Maund et al. [93] optimized and characterized a faithful culture model of the benign and malignant human prostate and found that the tissue slice cultures exhibited androgen dependence, appropriately undergoing ductal degeneration, reduced proliferation, and decreased prostate-specific antigen expression after androgen ablation. Furthermore, the tissue slice cultures showed cancer-specific reduction in androgen receptors and increased apoptosis after PL treatment [93]. Farrand et al. [94] investigated the influence of PL and CDDP (0 and 10 µM, 12 h) on mitochondrial fission in chemosensitive (OV2008) and chemoresistant (C13) OVCA cells. Both compounds increased mitochondrial fission and apoptosis in a concentration-dependent manner in OV2008 cells. Nonetheless, only PL had the same effect on C13 cells, suggesting that PL promotes apoptosis and induces mitochondrial fission in chemoresistant OVCA cells [94].

According to Fan et al. [15], PL application in HEPG2 and SMMC-7721 (hepatocellular carcinoma) cells led to an IC_50_ near 10 μM in both, and treatment of these cell lines at 10 μM of PL significantly inhibited cell proliferation. The study also found that PL promotes the expression of LINC01391. Furthermore, PL and overexpressed LINC01391 inhibited proliferation and invasion and promoted cell apoptosis. Mechanistically, LINC01391 inactivated the Wnt/β-catenin pathway by physical interaction with ICAT and upregulating its expression. Cheng et al. [95] demonstrated the effect of PL on circRNA expression in HCC cells and confirmed that PL suppresses HCC cell proliferation by circ-100338 function as competitive endogenous RNA.

Furthermore, Chen et al. [85] evaluated the anticancer effects of oxaliplatin combined with PL and found that 2.0 μM of PL as monotherapy does not significantly inhibit HCT-116 or LoVo (colorectal cancer) cells, although combining it with oxaliplatin sharply decreased cancer cell viability compared to monotherapies; nonetheless, it did not affect normal cells, since the IC_50_ values were 5.56, 7.30, and 20.7 μM in HCT-116, LoVo, and GES-1 cells, respectively. As for the pro-apoptotic effects, any agent alone only induced a slight increase in apoptosis, whereas a combination of oxaliplatin and PL at the same concentrations considerably increased the number of apoptotic cells. As for the in vivo effects, mice injected with HCT-116 cells were used, which when treated with the combination (5 mg kg^−1^ oxaliplatin and 2.5 mg kg^−1^ PL) reduced the weight, volume, and size of the tumors to a greater extent than monotherapy at the same concentrations. The combination also inflated the amounts of ATF4 and cleaved PARP, indicating that tumor cell apoptosis is associated with induced ER stress in vivo [85].

In addition, Machado et al. [81] investigated the response of the tumor cell line HCT 116, either wild type or deficient in Bax, p21, or p53 proteins, to PL, and its effect on different cell signaling pathways involved in apoptosis-dependent death was analyzed. This study revealed that PL has an IC_50_ in the HCT-116 cell line of 12.8 μM. Furthermore, the study demonstrated that PL induces cell death in HCT-116 cells, regardless of Bax, p21, and p53 status, but reduces cytotoxicity against the non-tumor cell line HEK-293 at ≥10 μM. Moreover, a PL concentration ranging from 0.5 to 2 μM significantly reduced the clonogenic capacity of HCT-116 cells. After treatment, the cells showed typical apoptotic features with 6 μM and 12 μMPL. At the highest tested concentration, late apoptosis and/or necrosis events ranged from 16.5–37.9% in HCT-116 cells. Furthermore, the study reported that PL induces G2/M cell cycle arrest in cells deficient in Bax, p21, and p53.

Furthermore, Zhou et al. [86] demonstrated that PL has a potential inhibitory effect on NSCLC cells in vitro and in vivo and found that PL at concentrations of 5 µM acts against cell proliferation in H23, HCC827, and H1975 (non-small-cell lung cancer) cells and that this effect is dose dependent. Since dysregulation of glycolysis is involved in human NSCLC tumorigenesis, with hexokinase 2 being the first limiting enzyme, it is required for tumor initiation and maintenance in mouse models of human lung cancer. Therefore, treatment with PL (2–10 μM) decreased the expression of HK2 but not HK1; PL was also shown to significantly suppress Thr473 phosphorylation in a dose-dependent manner (5–10 μM). The article demonstrates that PL promotes the activation of the intrinsic signaling pathway of apoptosis, including cytochrome c release and Bax translocation into mitochondria, with dose-dependent apoptosis. Furthermore, impaired phosphorylation of Akt at a 5–10 μM concentration was used for PL-mediated glycolysis suppression in NSCLC cells. Finally, PL was tested in vivo using a human NSCLC xenograft, with HCC827 or H1975 cells injected into mice. The study found that 10 mg/kg PL decreased the tumor size, volume, and weight and did not affect the body weight of the mice. Tissue samples from various organs revealed no pathological changes, and the data showed that treatment significantly suppresses p-Akt, p-S6, HK2, and Ki-67 [86].

In their study, Zhang et al. [82] explored the anticancer activity and mechanisms of action of PL against CRPC in terms of DNA damage and repair processes and demonstrated that PL has an IC_50_ of 6.75, 8.42, 8.73, and 68.62 μM in PC3 cells, DU145 cells (cells that are derived from bone and brain metastasis of CRPC), WPMY-1 cells, and LO2 cells. The results showed that PL exhibits anticancer activity in a dose-dependent manner (1, 2, and 4 μM) and is stronger against CRPC cells compared to taxol, cisplatin (DDP), doxorubicin (Dox), or 5-fluorouracil (5-FU), with fewer side effects in normal cells. In addition, 4.0 μM of PL induced a significantly lower migration rate in PL DU145 cells; PL concentrations of 1, 2, and 4 μM efficiently decreased FAK expression, especially p-FAK, supporting the conclusion that PL inhibits CRPC cell migration by suppressing FAK expression and distribution at the cell border. The researchers’ results also indicated that PL treatment (1, 2, and 4 μM) has no noticeable effect on cleaved PARP expression but slightly downregulates the expression of Bcl2, an important anti-apoptotic protein-associated protein, in PC3 cells. Nevertheless, PL significantly increased the content of cleaved PARP protein at 4.0 μM but had little effect on Bcl2 in DU145 cells. Furthermore, PL treatment (1, 2, and 4 μM) induced apoptosis in DU145 cells, with a concomitant increase in p53 expression. Finally, PL treatment triggered persistent DNA damage and caused strong DNA damage responses in CRPC cells [82].

Mohammad et al. [83] investigated the signaling mechanisms that contribute to PL-induced PDAC cell death and demonstrated that PL causes concentration-dependent cell death in MIA PaCa-2 and PANC-1 (pancreatic ductal adenocarcinoma (PDAC)) cells, such that MIA PaCa-2 cells are more sensitive to PL than PANC-1 cells at a 50% growth inhibition concentration (GI_50_) of 6.5 μM and 13.2 μM, respectively. PL significantly decreased GST activity in PDAC cells in a concentration-dependent manner (0, 10, or 100 μM) to 166.7, 64.2, and 21.5 mU/mL, respectively. Moreover, 10 μM of PL reduced the association of GSTP1 with JNK, which may lead to JNK activation. Furthermore, treatment with 10 μM of PDAC cells resulted in robust activation of JNK within 15 min, which decreased by 3 h in PANC-1 cells but remained elevated in MIA PaCa-2 cells; activation of c-Jun and ATF-2 also occurred. Furthermore, treatment with 10 μMPL for 6 h in MIA PaCa-2 cells resulted in positive regulation of various genes, including HMOX1 (31.7-fold increase), HSPA1A, CASP3, CDKN1A, MYC, and PIK3CG, and negative regulation of several genes, including BCL2, NFkB1, and AKT3. Still, treatment of PDAC cells with 10 μM resulted in robust cleaved caspase-3 and PARP. The authors also found that PL increases the nuclear expression of c-Myc protein in PDAC cells, and PL also demonstrated initial ERK activation at 15 min that decreased by 3 h in both cell lines, suggesting that PL leads to an early ERK signaling response suppressed over time. The results of this study show that PL causes dissociation of GSTP1 from JNK; robust JNK, c-Jun, and early ERK activation, followed by suppression; increased cleaved caspase-3 and cleaved PARP expression; and nuclear translocation of Nrf2 and c-Myc in PDAC cells. Gene expression analysis revealed that PL causes a >20-fold induction of heme oxygenase-1 (HO-1), which has been hypothesized to be a survival mechanism for PDAC cells under increased oxidative stress. Knockout of HO-1 resulted in PL-induced increased PDAC cell death under hypoxic conditions. Similarly, high concentrations of the HO-1 inhibitor, ZnPP (10 µM), sensitized PDAC cells to PL; however, lower concentrations of ZnPP (10 nM) and high or low concentrations of SnPP protected PDAC cells from PL-induced cell death. Interestingly, the JNK inhibitor significantly blocked PL-induced PDAC cell death, Nrf-2 nuclear translocation, and HMOX-1 mRNA expression. The results demonstrated that JNK signaling contributes to PL-induced PDAC cell death, while activating Nrf-2 transcription of HMOX-1 as a compensatory survival mechanism. These findings imply that elevating oxidative stress (with PL) while impairing antioxidant capacity (inhibiting HO-1) may be an effective therapeutic approach for PDAC [83].

PL induces CHOP, leading to the positive regulation of its targets Bim and DR5. Pretreatment with the ROS scavenger N-acetyl-cysteine abolishes PL-induced positive regulation of CHOP and its target genes, suggesting an essential role for ROS in PL-induced CHOP activation. Negative regulation of CHOP or Bim with siRNA efficiently attenuates PL-induced cell death, suggesting a critical role for CHOP in this cell death. Moreover, PL potentiates TRAIL-induced cytotoxicity in breast cancer cells via the positive regulation of DR5, as the knockdown of DR5 abolishes the sensitizing effect of PL on TRAIL responses [96].

In this context, the experiments of Gao et al. [87] investigated the antitumor effect of PL on CRC cells and revealed the underlying mechanism. PL at concentrations of 2, 5, and 10 μM decreased the cell viability of all these tested human colorectal cancer cells (HCT116, HT29, and SW620) in a dose-dependent manner. Notably, 2 μM of PL reduced the number of colonies by more than 40% in HCT116, HT29, and SW620 cells, whereas with 10 μM of PL, the number of colonies decreased by over 95% in all these CRC cells. Furthermore, treatment with 10 μM significantly decreased the population of histone H3 Ser10-positive cells, decreased the level of Cyclin D1 protein in CRC cells, and reduced AP-1 luciferase activity by over 70%; PL also induced cell cycle arrest in the G0/G1 phase in a dose-dependent manner (2–10 μM). Additionally, in a dose-dependent manner, the phosphorylation of EGFR at Tyr1068 was reduced; thus, the activity of kinases downstream of EGFR, Akt, and ERK1/2 was inhibited. Finally, by using a mouse xenograft model with HT29 cells, treatment with 10 mg/kg PL significantly decreased the tumor growth, weight, and volume [87].

## 3. Materials and Methods

This review was developed based on a survey of the literature on the antitumor activity of piplartine. The search was performed in the PubMed database and included studies published from 2012 to 2022. The studies’ eligibility and selection criteria were based on the following keywords: piplartine, piperlongumine, antitumor, cancer, and cytotoxicity. Only scientific publications published in the English language were selected.

## 4. Conclusions

The antitumor properties of piplartine have been proven in numerous animal and in vitro studies, showing strong evidence for its inhibitory effect against various types of tumors. Outstanding antitumor action was observed in experiments with gastric cancer, breast cancer, carcinoma of the parotid gland, and colon cancer. The experimental models used suggest several mechanisms of pharmacological action, such as caspases, NF-kB, MMP2, and Cyclin D1, resulting in cell death by apoptosis, necrosis, and oxidative stress, among other ways. Overall, the studies reported herein confirm the correlation of increased intracellular ROS and decreased inflammation in cancer cells, cell death pathway activation, and cell growth inhibition with the action of piplartine. Hence, the results provided serve as a reference to understand the mechanisms involved in the inhibition of tumor growth and the types of tumor cells sensitive to the antitumor action of this natural product. Piplartine has proved to be a promising antitumor agent and can be used as a prototype for planning and obtaining new drug candidates, aiming at the discovery of new chemical agents with therapeutic potential against cancer.

## Figures and Tables

**Table 1:** Antitumor effects of piplartine assessed in vitro or in vivo and its mechanisms.

Model	Specimen	Effect Observed EC_50_/IC_50_/GI_50,_ ROS		Cell Death Pathway	Reference
In vitro	HEPG2 SMMC-7721	10 μM (IC_50_) 10 μM (IC_50_)	nd	LINC01391 Wnt/β-catenin	[15]
	HeLa	16.3 μM (IC_50_)	nd	Foxo1, p21, p53, IκB-α	[23]
	MKN45 AGS	nd	nd	STAT3 JAK1/2	[26]
	786-O	5–10 μM (IC_50_)	nd	Sp1 Sp3 Sp4	[27]
	KKU-055 KKU-213 KKU-214 KKU-139 KKU-100 MMNK1 NIH3T3	4.2 μM (IC_50_) 5.2 μM (IC_50_) 6.2 μM (IC_50_) 8.8 μM (IC_50_) 15.9 μM (IC_50_) 5.7 μM (IC_50_) 12.7 μM (IC_50_)	Increase	BAX Caspase-3 p-JNK p-ERK FOXM1	[30]
	Daudi Raji Ramos DG-75	2.8 × 8.5 μM (IC_50_)	nd	NF-κB MYC p65	[32]
	NSCLC	0–15 μM (IC_50_)	nd	NF-κB	[33]
	HepG2	5 mM (IC_50_)	nd	AMPK	[36]
	INT-407 HCT-116	13 and 9 μM (IC_50_) 8 and 6 μM (IC_50_)	Increase	P53 P21 Bax SMAD4 BCL2 SURVIVIN	[39]
	WERI-Rb Y79	10 μM (IC_50_)	Increase	Caspase-3 PARP	[41]
	ICP HeLa	15 μM (IC_50_) 2.7 ± 0.1 μM (IC_50_)	nd	nd	[45]
	A375 A875 B16-F10	0.6 mM (IC_50_) 2.5 mM (IC_50_) 2.5mM(IC_50_)	Increase	Caspase-3 Bax Bcl-2 p-JNK	[48]
	H1703 HeLa	2.8 μM (EC_50_) 7.1 μM (EC_50_)	Increase	nd	[60]
	Bel7402 Bel7402/5-Fu HepG2 HGC27	5.6 μM (IC_50_) 8.4 μM (IC_50_) 6.8 μM (IC_50_) 8.2 μM (IC_50_)	Increase	nd	[61]
	MDA-MB-231 MCF-7 A549 KB KB-VIN	3.72 μM (IC_50_) 4.4 μM (IC_50_) 3.2 μM (IC_50_) 1.67 μM (IC_50_) 7.85 μM (IC_50_)	Increase	TrxR TrxR1 Bcl-2 Bcl-xL LC3-II Beclin-1	[62]
	MDA-MB-231 A549 HGC27 HT-29 HepG2	6.53 μM (IC_50_) 5.31 μM (IC_50_) 8.15 μM (IC_50_) 6.25 μM (IC_50_) 7.02 μM (IC_50_)	Increase	TrxR	[63]
	MIAPaCa-2	8 μM (IC_50_)	Increase	Ferroptosis	[64]
	HGC27 SGC7901 MCF7 HCT116	7.53 μM (IC_50_) 5.79 μM (IC_50_) 4.68 μM (IC_50_) 6.61 μM (IC_50_)	Increase	TrxR Bax PARP Bcl-2 H2AX(S139ph) p53 p-p53	[65]
	CT26 DLD-1	15.98 μM (IC_50_) 11.20 μM (IC_50_)	Increase	TrxR	[66]
	A549 HepG2 HT-1080 WI-38	10.17 ± 0.18 µM (IC_50_) 8.08 ± 0.30 µM (IC_50_) 5.55 ± 0.66 µM (IC_50_) 60 µM (IC_50_)	Increase	TrxR	[67]
	HeLa A549	4.4 ± 0.1 μM (IC_50_) 11.7 ± 2.0 μM (IC_50_)	Increase	nd	[68]
	HCC	10–20 μM (IC_50_)	Increase	PERK/Ire1α/GRP78 p38/JNK/Erk C/EBP	[69]
	nd	nd	nd	NF-κB IκBα Bcl-2 Bcl-xL c-IAP-1 c-IAP-2 Survivin c-Myc Cyclin D1 COX-2 IL-6 ICAM-1 CXCR-4 VEGF	[70]
	AMC-HN9 AMC-HN3 UMSCC-1	10 μM (IC_50_)	Increase	p53 PARP PUMA	[71]
	AsPc-1 Panc-1 HPAC L3.6PL COLO-357	5-10 µM (IC_50_)	nd	STAT3 Mcl-1 Bcl-2 PARP	[72]
	hf5281	5 μM (IC_50_)	nd	nd	[73]
	U937	20 µM	nd	p-mTOR p-p38 Caspase-3	[74]
	A549 HCT-116 MDA-MB-231 SK-Hep-1 Saos-2	6.84 µM (IC_50_) 7.34 µM (IC_50_) 10.6 µM (IC_50_) 13.3 µM (IC_50_) 9.49 µM (IC_50_) 7.31 µM (IC_50_)	nd	nd	[75]
	HR-deficient brca1 HR-deficient brca2	0.8–1.2 μM (IC50) 1.2–1.6 μM (IC50)	nd	DNA double-strand breaks	[76]
	MCF-7	5 μM (IC_50_)	nd	nd	[77]
	A549 HCT116 ZR-75-30 MDA-MB-231 MRC-5	22.85 μM (IC_50_) 6.04 μM (IC_50_) 5.86 μM (IC_50_) 8.46 μM (IC_50_) 35.04 μM (IC_50_)	nd	nd	[78]
	HT-29 HCT 116	10.1 μM (IC_50_) 6.4 μM (IC_50_)	nd	p-ERK	[79]
	NCI-H929	1–5 μM (IC_50_)	nd	Caspase-3 Caspase -9 Caspase -8 Bcl-2	[80]
	HCT-116	12.8 μM (IC_50_)	nd	Bax p21 p53	[81]
	PC3 DU145 WPMY-1 LO2	6.75 μM (IC_50_) 8.42 μM (IC_50_) 8.73 μM (IC_50_) 68.62 μM (IC_50_)	nd	Bcl2 p53	[82]
	MIA PaCa-2 PANC-1	6.5 μM (GI_50_) 13.2 μM (GI_50_)	Increase	GST GSTP1/JNK Caspase-3 PARP JNK c-Jun ERK HMOX-1	[83]
	MCF-7 Jurkat	1.2 ± 0.6 μM (IC_50_) 1.4 ± 0.3 μM (IC_50_)	nd	Tubulin depolymerization	[22]
In vitro/In vivo	T24 BIU-87 EJ	1–20 µM (IC_50_)	nd	Slug β-catenin ZEB1 N-cadherin Claudin-1 ZO-1	[31]
	U2OS MG63	10.02 μM (IC_50_) 8.387 μM (IC_50_)	nd	JAK2 STAT3	[37]
	A549	5 mg/kg (IC_50_)	nd	TGFBR1	[38]
	SAS CGHNC8	2.5 and 5.0 µM (IC_50_)	nd	Oct-4 NANOG SOX2 SRY-Box 2 POU class 5 homeobox 1 Nanog homeobox	[40]
	MDA-MB-231 BT-549 Hs578T	10 µM (IC_50_)	Increase	MMP2 MMP9 IL-6 ZEB1	[42]
	U87MG HCT-116 A549 lK562 HT-29 SW620 HCT-8 CCD-841	16.15 ± 0.81 µM (IC_50_) 8.17 ± 0.26 µM (IC_50_) 15.22 ± 1.20 µM (IC_50_) 5.09 ± 0.10 µM (IC_50_) 0.54 ± 0.04 µM (IC_50_) 0.87 ± 0.06 µM (IC_50_) 1.21 ± 0.08 µM (IC_50_) 51.55 ± 2.08 µM (IC_50_)	Increase	nd	[43]
	T24 BIU-87 EJ	10-20 μM (IC_50_)	Increase	ROS Erk PKC	[31]
	HeLa MCF-7 MGC-803	12.89 and 10.77 μM (IC_50_) 13.39 and 11.08 μM (IC_50_) 2.55 and 9.72 μM (IC_50_)	nd	Caspase-9 Caspase-3 Akt	[47]
	OVCAR-3 A2780	4.59 μM (IC_50_) 10 μM (IC_50_)	Increase	nd	[84]
	HCT-116 LoVo GES-1	5.56 μM (IC_50_) 7.30 μM (IC_50_) 20.7 μM (IC_50_)	nd	ATF4 PARP	[85]
	H23 HCC827 H1975	5 µM (IC_50_) 5–10 µM (IC_50_) 5–10 µM (IC_50_)	nd	HK2 Cytochrome C Bax Thr473 p-Akt p-S6 HK2 Ki-67	[86]
	HCT116 SW620 HT29	2.5–10 μM (IC_50_)	nd	Cyclin D1 G0/G1 EGFR Akt ERK1/2	[87]
In vivo	MPC cell	5–10 μM (IC_50_)	Increase	ERK-1/2, p38α, NK1/2/3	[17]
	A549 anchorage dependent A549 anchorage independent	3.06–52.44 μM (IC_50_) 0.86–11.66 μM (IC_50_)	nd	pSTAT3 mRNA	[25]
	SF-295 HCT-8	0.8 μg/mL (IC_50_) 0.7 μg/mL (IC_50_)	nd	nd	[34]
	SGC-7901	4 mg/kg (IC_50_) 12 mg/kg (IC_50_)	nd	nd	[35]
	Colon carcinogenesis induced by DMH + DSS	3.6 mg/kg (IC_50_)	nd	Ras PI3K/Akt Akt/NF-κB c-Myc Cyclin D1 Bcl-2 Caspase-3	[44]
	DMH/DSS-induced colon cancer	3.6 mg/kg (IC_50_)	nd	NF-κB COX-2 NF-κB β-catenin IL-6 JAK2 STAT3 Jagged-1 Notch-1 MMP-9 Vimentin TCF/ZEB	[46]
	A375 HeLa HPDE LO-2 HK-2 MCF10a	2 µM (IC_50_) 4 µM (IC_50_) 8 μM (IC_50_) 8 µM (IC_50_) 8 µM (IC_50_) 8 µM (IC_50_)	Increase	Bcl-2 NK cell	[88]
	nd	10 μM (IC_50_)	Increase	GADD45α p21 Cyclin B1 cdc2 Caspase-3 Caspase-7 Caspase-9 PARP	[89]
	LN229 U87 8MG	20 μM (IC_50_)	Increase	JNK p38	[90]

GI_50_: 50% growth inhibition concentration; EC_50_: 50% effective concentration; IC_50_: 50% inhibitory concentration; ROS: intracellular reactive oxygen species; nd: not determined.

## Data Availability

Data sharing is not applicable.

## Abbreviations

5-FU	5-fluorouracil
ABC	activated B-cell receptor
ACC	acetyl-CoA carboxylase
AML	acute myeloid leukemia
AMPK	AMP-activated protein kinase
ATF4	activating transcription factor-4
ATO	arsenic trioxide
BCNU	1,3-bis(2-chloroethyl)-l-nitrosourea
BM	bone marrow
CCA	cholangiocarcinoma
CDDP	cisplatin: cis-diamminedichloroplatinum(II)
CHOP	C/EBP homologous protein
CK18	cytokeratin 18
CN-A	cotylenin A
COX-2	cyclooxygenase 2
CRC	colorectal cancer
CRPC	metastatic castration-resistant prostate cancer
CXCR-4	CXC chemokine receptor 4
DCFH-DA	2′-7′-dichlorofluorescein diacetate
DDP	cisplatin
DDS	dextran sulphate sodium
DLBCL	diffuse large B-cell lymphoma
DMH	1,2-dimethylhydrazine
DMSO	dimethylsulfoxide
DOX	doxorubicin
EGFR	epidermal growth factor receptor
EMT	epithelial–mesenchymal transition
ER	endoplasmic reticulum
Erk	extracellular-signal-regulated kinase
ERK	extracellular-signal-regulated kinases
FAK	focal adhesion kinase
FOXM1	Forkhead Box M1
FOXO3A	transcription factor forkhead box O 3A
GBM	glioblastoma multiforme
GC	gastric cancer
GI_50_	50% growth inhibition concentration
GLO1	glyoxalase I
GSH	glutathione
GSSG	glutathione disulfide
GSTO1	glutathione S-transferase omega 1
GSTP1	glutathione S-transferase pi 1
HCC	hepatocellular carcinomas
HFA	hollow fiber assay
HGG	high-grade glioblastoma
HNC	head and neck cancer
HO-1	heme oxygenase-1
HR	homologous recombination
HUVECs	human umbilical vein endothelial cells
IC_50_	half maximal (50%) inhibitory concentration
ICAM-1	intercellular adhesion molecule-1
ICAT	T-cell factor
iCP	human 20S immunoproteasome
IKK	IκB kinase
IL-6	interleukin-6
IL-8	interleukin-8
IκB	inhibitory kappa B
JAK2	Janus kinase 2
JNK	c-jun N-terminal kinase
LASA	lipid-associated sialic acid
LDH	lactate dehydrogenase
MEF	embryonic fibroblast
MM	multiple myeloma
MMP-9	metalloproteinase-9
MPC cells	mouse pheochromocytoma cells
MTT	3-(4,5-dimethylthiazol-2-yl)-2,5-diphenyltetrazolium bromid assay
MYC	mielocitomatose oncogênica
NADPH	nicotinamide adenine dinucleotide phosphate
NF-κB	nuclear transcription factor
NF-κB/MYC	nuclear factor kappa-B/myelocytomatosis oncogene
NK	natural killer
Nrf2	nuclear factor-erythroid-2-related factor-2
NSCLC	non-small-cell lung cancer
NSCs	normal neural stem cells
OS	osteosarcoma
OVCA	chemoresistant ovarian cancer
PARP	poly(ADP-ribose) polymerase
PDAC	pancreatic ductal adenocarcinoma
p-ERK	phosphorylated ERK
p-JNK	p-Jun N-terminal kinase
PL	piplartine
PRDX4	peroxiredoxin 4
Prx-2	peroxiredoxin-2
RIP1	receptor-interacting protein kinase 1
ROS	reactive oxygen species
siRNA	small interfering RNA
SOCS3	cytokine signaling 3
STAT3	signal transducer and activator of transcription3
TCF/ZEB	T-cell factor/zinc finger E-box-binding homeobox protein
TGFBR1	transforming growth factor beta type I receptor
TNF-α	tumor necrosis factor alpha
Trx	thioredoxin
TrxR	thioredoxin reductase
TSA	total sialic acid
ZnPP	zinc protoporphyrin
ZO-1	zonula occludens-1
